# HAIviz: an interactive dashboard for visualising and integrating healthcare-associated genomic epidemiological data

**DOI:** 10.1099/mgen.0.001200

**Published:** 2024-02-15

**Authors:** Budi Permana, Patrick N. A. Harris, Leah W. Roberts, Thom Cuddihy, David L. Paterson, Scott A. Beatson, Brian M. Forde

**Affiliations:** ^1^​ School of Chemistry and Molecular Biosciences, The University of Queensland, St Lucia, Queensland, Australia; ^2^​ University of Queensland Centre for Clinical Research, Royal Brisbane and Women’s Hospital, Herston, Queensland, Australia; ^3^​ Herston Infectious Diseases Institute, Metro North Health, Queensland, Australia; ^4^​ Pathology Queensland, Central Laboratory, Royal Brisbane and Women’s Hospital, Herston, Queensland, Australia; ^5^​ Centre for Immunology and Infection Control, Queensland University of Technology, Brisbane, Queensland, Australia; ^6^​ ADVANCE-ID, Saw Swee Hock School of Public Health, National University of Singapore, Singapore, Singapore; ^7^​ Infectious Diseases Translational Research Programme, Yong Loo Lin School of Medicine, National University of Singapore, Singapore, Singapore; ^8^​ Australian Infectious Disease Research Centre, The University of Queensland, St Lucia, Queensland, Australia

**Keywords:** genomic epidemiology, healthcare-associated infections, pathogen genomic surveillance, visualisation tool

## Abstract

Existing tools for phylogeographic and epidemiological visualisation primarily provide a macro-geographic view of epidemic and pandemic transmission events but offer little support for detailed investigation of outbreaks in healthcare settings. Here, we present HAIviz, an interactive web-based application designed for integrating and visualising genomic epidemiological information to improve the tracking of healthcare-associated infections (HAIs). HAIviz displays and links the outbreak timeline, building map, phylogenetic tree, patient bed movements, and transmission network on a single interactive dashboard. HAIviz has been developed for bacterial outbreak investigations but can be utilised for general epidemiological investigations focused on built environments for which visualisation to customised maps is required. This paper describes and demonstrates the application of HAIviz for HAI outbreak investigations.

## Data Summary

HAIviz is a cross-platform web-based visualisation application developed using JavaScript. It is an open-source application distributed under a GPLv3 license. The complete source code and build application are available in GitHub (https://github.com/nalarbp/haiviz). The online version of HAIviz is hosted at https://haiviz.fordelab.com. Supplementary material consisting of supplementary methods, tables, and a quick start guide file are available with the online version of this article.

**Impact Statement**

Whole genome sequencing (WGS) is establishing itself as the optimal method for unravelling transmission dynamics of hospital-associated infections (HAI). The key to effectively harnessing WGS for infection prevention and control (IPC) lies in seamlessly merging intricate genomic insights with epidemiological data and presenting this amalgamation succinctly to clinicians. This paper describes HAIviz, a versatile, customisable tool designed for infection control management and visualisation. It seamlessly integrates WGS data with clinical, spatial, and temporal metadata within an intuitive, all-in-one interactive dashboard. HAIviz equips clinical staff with the power to vividly explore and contextualise emerging and ongoing outbreaks, enabling swift targeted interventions. This paper illustrates the practicality of thorough case studies of previously described outbreaks and confirms its capacity to deliver comparable results. HAIviz, has been successfully deployed by IPC teams to support clinical management of HAIs and it holds the promise of widespread adoption in diverse settings, encompassing clinical pathogen surveillance, as well as applications in public health and industrial domains.

## Introduction

Pathogen genomics has emerged as a valuable tool for outbreak control and pathogen surveillance in healthcare settings [1–4]. The analysis of genome sequences enables detailed characterisation of pathogen relatedness, facilitating more accurate identification of outbreak clusters [5–7]. Furthermore, results from genomic analysis, such as single nucleotide polymorphisms (SNPs), can be integrated with epidemiological data to predict transmission routes and determine the outbreak’s origin [8–10].

The challenge of effectively integrating and visualising genomics alongside associated epidemiological data in a manner that is clinically meaningful remains challenging. During an active outbreak, infection control teams must carefully evaluate genomic relationships, temporal and geographic factors, and other related data to establish epidemiological connections among cases.

The development of phylogeographic and genomic epidemiological visualisation tools, such as Microreact [11], Pathogenwatch [12], and Nextstrain [13], has provided a valuable resource for visualising and creating interactive connections between phylogenetic trees, timelines, and geographical maps, thereby providing a powerful means to understand the dissemination of pathogens. However, their effectiveness is more pronounced in the context of national or global outbreaks, and their utility diminishes in the case of localised outbreaks, such as those confined within healthcare facilities or specific wards within these facilities. These ‘local’ outbreaks typically spread through direct patient-to-patient contact, via healthcare personnel and fomites, or through contact with reservoirs within the local environment. For example, outbreaks of hospital-acquired Legionnaires' disease have been linked to water distribution systems (WDSs) [14–17], and patient-to-patient transmissions are common in nosocomial outbreaks [18, 19].

The utilisation of a site-specific map delineating, for example, WDS-related facilities or the specific locations of beds within hospital wards, can prove extremely advantageous in the process of pinpointing origins of contamination and tracing routes of transmission. It is worth noting that these functionalities, which encompass the tracking of in-hospital patient movements (such as changes in wards or beds), are limited when used with current standalone tools for genomic epidemiological visualisation.

To address this gap, we have developed an interactive web-based visualisation tool called the Healthcare-Associated Infection Visualisation Tool (HAIviz). HAIviz empowers users to craft personalised maps and visualise essential clinical metadata, such as patient mobility, sample timelines, phylogenetic trees, and transmission networks, all within a unified and interactive web dashboard. As a result, HAIviz serves as both an exploratory instrument for collaborative data analysis and a platform for managing and reporting on infection control. In this context, we present an overview of HAIviz’s implementation and functionality for localised investigations into nosocomial outbreaks.

## Implementation

### Tool overview

**Fig. 1.**
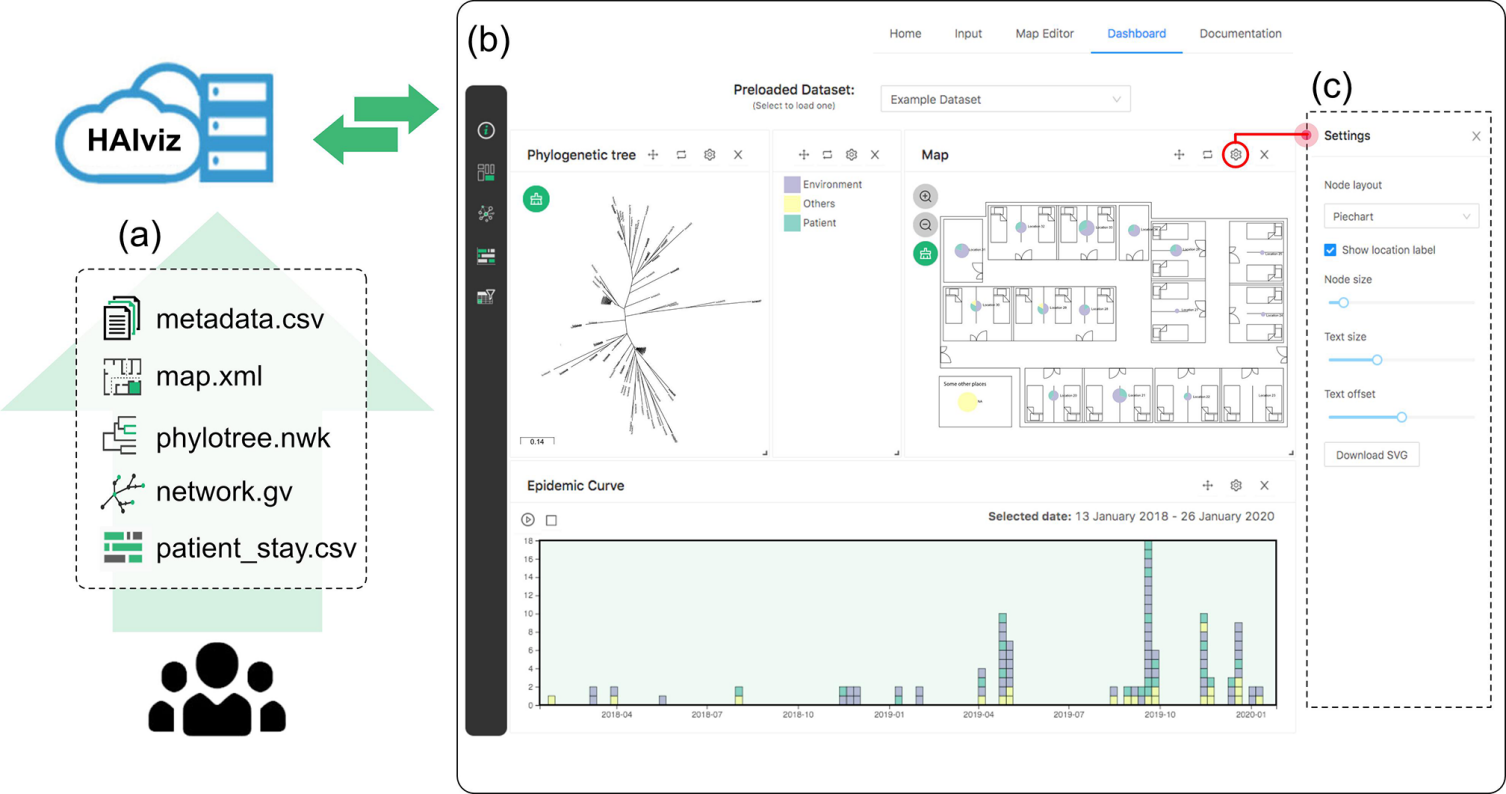
The schematic workflow of using HAIviz. (**a**) Users load input files into HAIviz. (**b**) HAIviz processes input data and renders a visualisation dashboard. (**c**) Setting panel, displaying related configuration and button for each associated window in the dashboard.

HAIviz is a client-side web-based visualisation tool built with React.js. Using HAIviz, users can easily create an interactive genomic epidemiological visualisation dashboard using a web browser (Fig. 1). Through a drag-and-drop interface, users can display and integrate interactive timelines, a customised map, metadata table, phylogenetic tree, and transmission network. All visualisation processes are performed locally in the user’s browser with no data uploaded to the server, ensuring the safety of private data. The tool is free to use online at https://haiviz.fordelab.com and the bundle application is available at https://github.com/nalarbudi/haiviz/ for self-hosting or offline use.

### User interface

The HAIviz user interface (UI) consists of five integrated pages: *Home, Input, Map editor, Dashboard, and Documentation*. The *Home* page serves as the landing page and displays preloaded datasets installed within HAIviz. The *Input* page contains input placeholders for users to drag and drop their input files. The *Map editor* page allows users to create or update a HAIviz map using standard images, such as joint photographic experts group (JPEG) or portable network graphic (PNG) image. The *Dashboard* page enables users to generate and manage interactive visualisation windows. Finally, the *Documentation* page provides users a quick start guide, usage examples, and input file templates.

The UI were developed using Ant Design UI for React.js (https://ant.design/). The grid functionality on the dashboard is implemented using the react-grid-layout (https://github.com/react-grid-layout). The phylogenetic tree is rendered using Phylocanvas v2.8.1 (https://phylocanvas.org/) and the transmission network is visualised using Cytoscape.js [20].

### Input and output files

HAIviz accepts up to five input files: a metadata table, a location map, a phylogenetic tree, a transmission network, and a patient stay timeline (Fig. 1a). The *metadata input file* is a comma-separated value (CSV) table file with three mandatory columns: id, date, and location. Optional columns can be included for additional data such as sample source, patient id, or user-defined colour. The *map input file* is an extensible markup language (XML) file containing map and location data. This map is specific to HAIviz and can be created in the *Map editor* page. The *phylogenetic tree input file* is a Newick formatted phylogenetic tree containing taxa names and branch lengths.

The *transmission network input file* is a DOT formatted graph file [21] consisting of nodes, edges, and their attributes. The node *name* attribute is mandatory for integration with the metadata. Edge attributes, such as *direction*, *weight*, *colour*, and *style* are optional for edge customisation. The DOT file allows users to present a diverse set of diagrams, including transmission or clustering networks produced by genomic epidemiological and graph-based tools such as Outbreaker2 [22], iGraph [23], and GraphSNP [24], as well as user-refined diagrams that might be created by manual inference or drawing. The *patient stay timeline input file* is a CSV file containing four mandatory columns: patient id, patient location, patient stay start date, and patient stay end date. To link the timeline with metadata, an additional patient id column in the metadata must be added. Detailed specifications for these input files are described in supplementary file S2. The input template and example files are available in the *Documentation* page.

Once the input is loaded and visualisation is rendered, users can download the visualisation result from each visualisation window using the download button from the window setting (Fig. 1c).

### Preloaded datasets

HAIviz is packed with preloaded datasets for project showcase and demonstration purposes. The datasets are a collection of bundled input files, which described by a configuration file to make it readily available in HAIviz *Home* page. Users can configure this file and set their datasets when they self-host or use HAIviz offline. This feature allows for seamless integration with tools or pipelines to direct input files into HAIviz for continuous use and updates. Detailed instructions on how to customise the preloaded datasets are provided in the *Documentation* page.

## Results

To demonstrate HAIviz’s capabilities, we have developed illustrative visualisation dashboards utilising data from three previously published outbreak studies [6, 25, 26]. These demonstration datasets have been preloaded into HAIviz and are readily accessible on the web page. Detailed methodologies employed to construct these datasets can be found in supplementary file S1.

### Using hospital ward layout to visualise the outbreak of carbapenem-resistant *Acinetobacter baumannii* in a Brisbane hospital

**Fig. 2.**
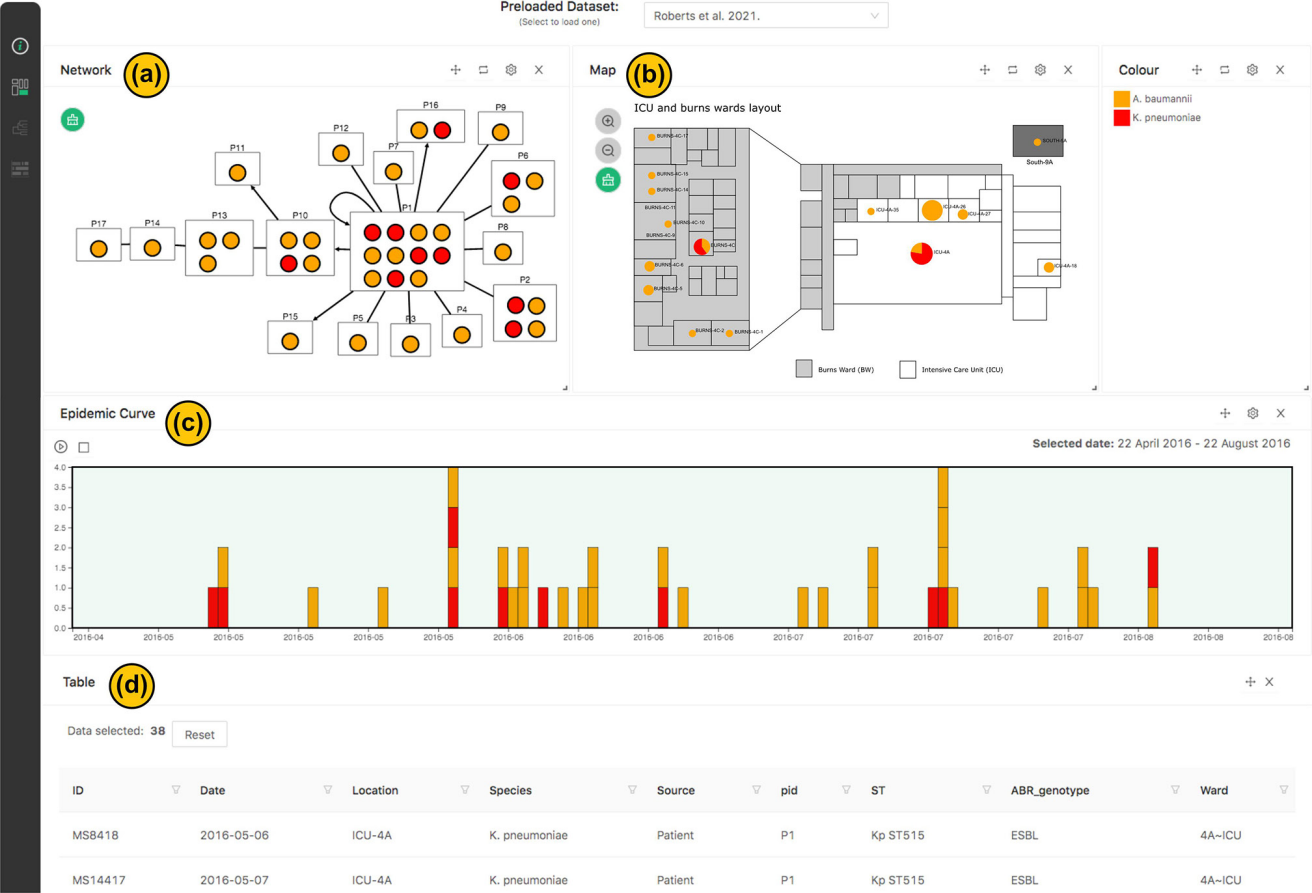
HAIviz dashboard visualising a polymicrobial outbreak at a Brisbane intensive care unit. (**a**) The predicted transmission network, showing the relationship between patients colonised by carbapenem-resistant *A. baumannii* and ESBL-producing *K. pneumoniae* [25]. Directed edges (lines with arrows) represent transmission directionality inferred by the accumulation of the SNPs between patients’ isolates, while the undirected edges (straight lines) show isolate relationships where the directionality cannot be inferred (i.e. they are identical at the core genome level). (**b**) The map window, displaying the number of isolates identified in the ICU and Burns wards. (**c**) The epidemic curve, showing sample collection dates. (**d**) The table window, showing isolate’s metadata. Colour indicates species.

The initial dashboard example (Fig. 2) is drawn from a genomic surveillance study conducted by Roberts *et al*. [25]. The study investigated a polymicrobial multidrug-resistant outbreak that occurred within a tertiary hospital in Brisbane. Clustering was inferred using core-genome SNP distances with <10 SNPs indicating direct patient-to-patient transmission. Transmission direction was manually inferred based on the accumulation of SNPs and the date of isolation. Although this outbreak involved five distinct bacterial species, the primary focus was on the transmission of carbapenem-resistant *Acinetobacter baumannii* (CR-Ab) and extended-spectrum beta-lactamase (ESBL)-producing *Klebsiella pneumoniae*. The investigation utilised whole genome sequencing (WGS) in conjunction with epidemiological data to decipher the transmission patterns among patients, with findings communicated to both clinicians and the infection control team.

HAIviz enabled us to visually represent and interlink various elements of the outbreak, including the predicted transmission routes (Fig. 2a), the hospital’s ward layout (Fig. 2b), the timeline of sampling (Fig. 2c), and the relevant metadata (Fig. 2d). HAIviz’s unique feature allowed for the easy use of the floorplan image as a map, eliminating the need for geolocation data. With the animation feature, we were able to showcase the progressive spread of CR-Ab and co-transmission of ESBL-producing *K. pneumoniae* within the intensive care unit (ICU) and the Burns wards. This outbreak demonstrated the necessity for a visualisation tool to better communicate genomic results to major stakeholders within the hospital. As such, a key feature of HAIviz enables the users to download or record visualised and animated results for inclusion in reports that are often necessary during ongoing infection control management.

### Visualising transmissions network of *Enterococcus faecium* ST78 outbreak in a Brisbane hospital

**Fig. 3.**
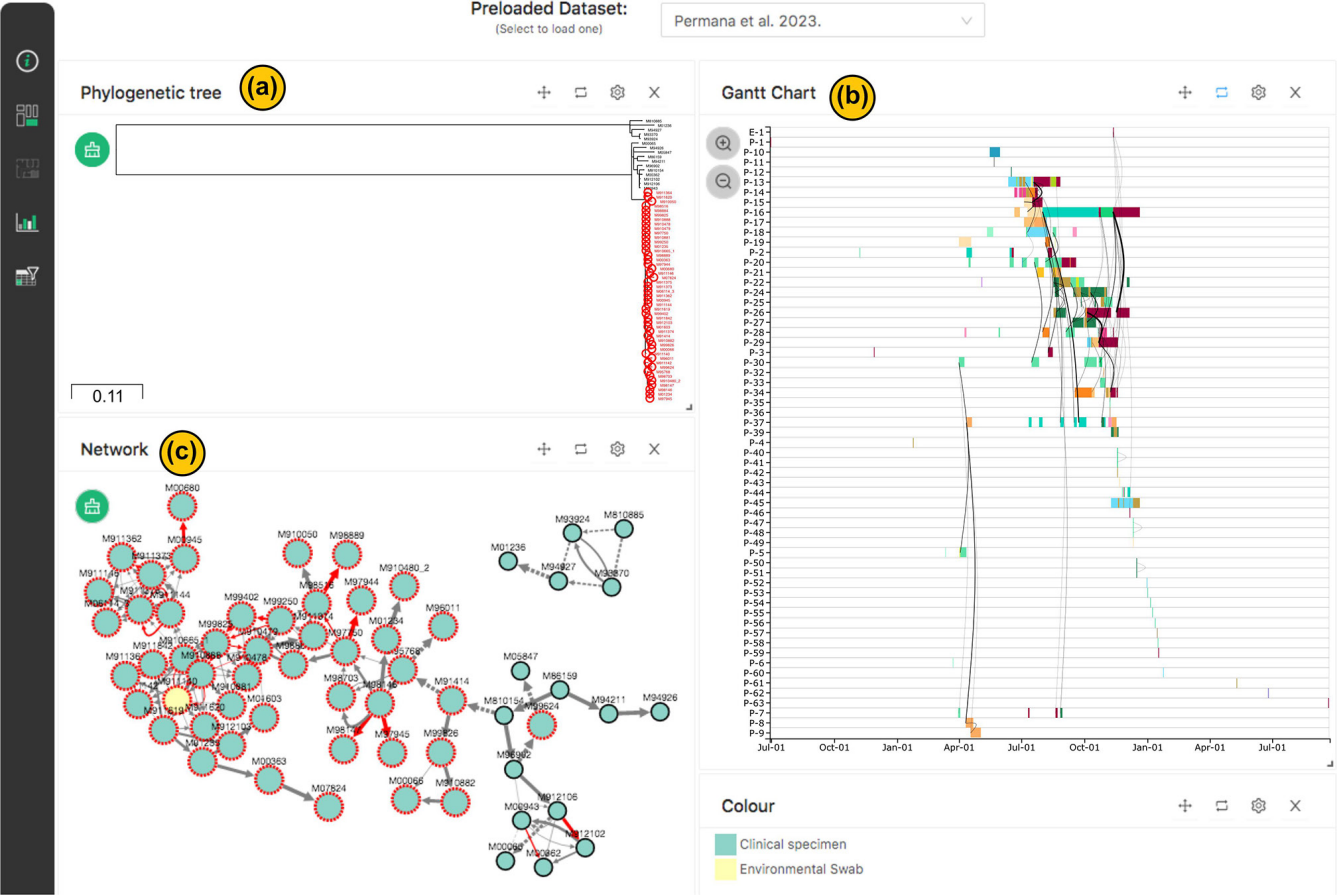
HAIviz dashboard visualising the outbreak of vancomycin-resistant *E. faecium* ST78 in a tertiary hospital in Brisbane [6]. (**a**) The phylogenetic tree, showing two distant lineages of VREfm ST78. The selected nodes were highlighted in red. (**b**) Gantt chart, showing the history of patient stays during the outbreak. Each rectangle represents one continuous patient stay coloured by location. Lines connecting the rectangles indicates overlapping on location (e.g. shared a same ward). Line width denotes duration (number of day) of the overlapping period. (**c**) Transmission network, displaying complex transmissions between isolates. Red-outlined nodes indicate the selected isolates. Edge denotes transmission direction scaled by transmission probability. Dashed line indicates indirect transmission between isolates from different cluster. Red line illustrates transmissions supported by ward sharing. Node’s colour denotes sample type.

Our second dashboard example (Fig. 3) stems from our genomic study investigating an outbreak of Vancomycin-resistant *Enterococcus faecium* (VREfm) ST78 within a large tertiary hospital in Brisbane [6]. This study combined SNP-typing with patient movement data to reveal clusters and transmission links between suspected outbreak isolates. Core genome SNPs were again used to define relationships, with a threshold of 20 SNPs used to infer clustering of closely related isolates. Transmission pathways were constructed using outbreaker2 [22], which using SNPs distance and temporal (collection dates) data, implements a Bayesian framework to infer the direction of transmission. HAIviz played a pivotal role in presenting and interconnecting the results, facilitating their interactive incorporation into infection control reports.

The VREfm ST78 study isolates revealed two distinct lineages, as evident in the phylogenetic tree (Fig. 3a). Notably, the outbreak clusters were predominantly located within one lineage, displaying minimal genomic diversity (Fig. 3a). The network visualisation further unveiled a complex web of transmission routes within the clusters, with a low probability of directionality (Fig. 3b). The representation of patients’ stay histories (Fig. 3c) highlighted instances of shared ward occupancy among patients. This finding strongly suggested that transmissions were likely occurring through patient-to-patient interactions and environmental reservoirs.

### Using interactive features to highlight outbreak of *Klebsiella pneumoniae* ST15 and ST1559 in a Nepal hospital

The third illustrative dashboard is derived from a genomic epidemiological study conducted by Chung and colleagues at a hospital in Nepal [26] and offers insights into the dynamics of *K. pneumoniae* outbreaks. This study leveraged genomics to characterise outbreaks attributed to two distinct *K. pneumoniae* lineages, namely sequence type (ST)15 and ST1559. Temporal and genetic relatedness were used to define suspect outbreak clusters, which were then reconstructed using maximum-likelihood (ML) phylogeny. We created an interactive version of the study results by reconstructing the phylogenetic tree and transforming the hospital layout into a HAIviz map. Using HAIviz, we have interconnected these findings, enabling the depiction of spatial, temporal, and genomic relationships between ST15 and ST1559.

**Fig. 4.**
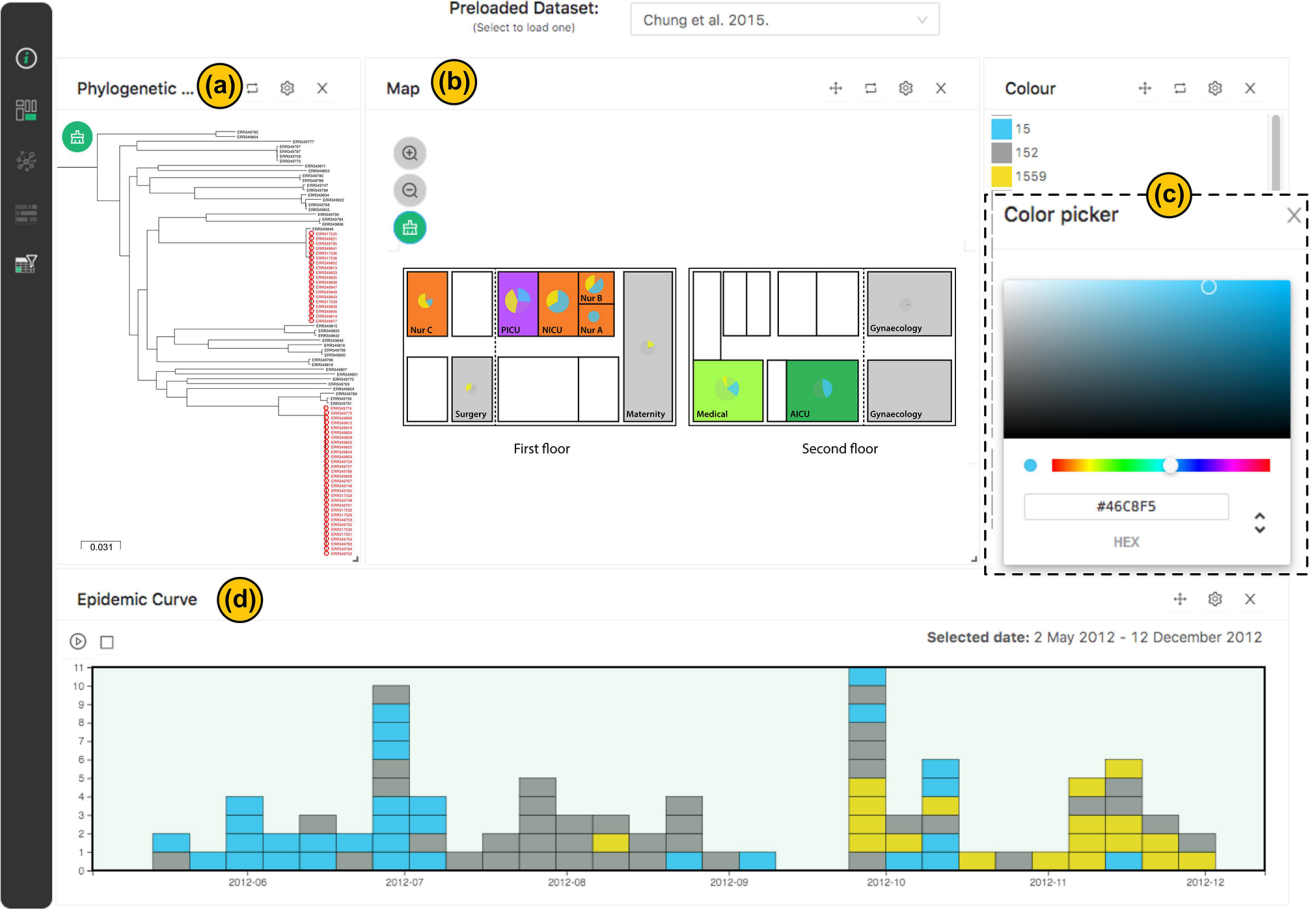
HAIviz visualises the outbreak of multidrug-resistant *Klebsiella pneumoniae* at a major hospital in Nepal [26]. (**a**) The phylogenetic tree. Red-coloured branches indicates the outbreak-causing sequence type (ST) 15 and 1559. (**b**) The hospital layout, adapted from Chung *et al*. [26], showing the composition of STs in each location. (**c**) Colour picker, allowing users to interactively change the colour (ST15: blue, ST1559: yellow). (**d**) The epidemic curve, displaying temporal differences in ST15 and ST1559 outbreaks.

Through the dashboard, we have applied distinctive colours to highlight these STs (Fig. 4c). The coloured epidemic curve intuitively conveys temporal variations between the STs (Fig. 4d), while their evolutionary relationship is clearly illustrated in the phylogenetic tree window (Fig. 4a). Employing animation, we have effectively visualised the movement of these isolates over the hospital map, emphasising the propagation of ST15 across the Neonatal Intensive Care Unit (NICU), Paediatric Intensive Care Unit (PICU), as well as Nurseries A and B wards (Fig. 4b).

### Feature comparison with the existing tools

To demonstrate the novelty of our platform, we conducted a comparative analysis of HAIviz’s features with those of Microreact, Pathogenwatch, and Nextstrain. While HAIviz does share several common visualisation functions with these tools, particularly with Microreact, it incorporates critical features that facilitate outbreak management and patient contact tracing, both within and across healthcare facilities. These features encompass a fully customizable mapping tool (including floor plans, water distribution schematics, and photographs) and comprehensive patient movement data (comprising information on visited wards, timeframes, and co-location data). These unique capabilities are notably absent in existing tools like Microreact, Pathogenwatch, or Nextstrain. For a comprehensive breakdown of feature descriptions and a detailed comparison, please refer to the supplementary table S2 provided in supplementary file S1.

### Browser compatibility and performance

HAIviz has been tested and is compatible with the recent version of Mozilla Firefox, Google Chrome, Apple Safari, Opera and Microsoft Edge. The performance of HAIviz is conditional upon the size of the dataset being explored, with larger datasets consuming more memory and CPU (supplementary figure S1). Our performance tests indicate that when all visualisation windows are displayed, visualising a dataset not exceeding 500 samples results in optimal resource usage. Notably, the network window is the most resource intensive. Therefore, we discourage users from using this window when loading larger datasets (e.g. > 500 samples), as it could trigger unresponsiveness and/or generate overpopulated and uninformative graphs. Detailed reports on both the browser compatibility and performance tests are provided in supplementary table S1 and supplementary table S3, respectively.

## Discussion

Current tools for phylogeographic and epidemiological visualisation primarily cater to macro-geographic perspectives of epidemic and pandemic transmission and offer limited support for visualising outbreaks within healthcare environments. Here, we introduce HAIviz, a bespoke visualisation tool that empowers users to represent outbreaks on a non-geographic map, visualise patient mobility, and seamlessly integrate this information with other genomic epidemiological data, all within a single interactive web dashboard. This dashboard serves as an ideal solution for reporting and conveying information about healthcare-associated outbreaks and is equally applicable for other pathogen surveillance applications.

Utilising a common image format such as PNG or JPEG to construct a map offers a significantly higher degree of flexibility and simplicity in comparison to relying on geographical coordinates. Converting a building floorplan into a geography-based map, typically requiring the use of formats like shapefiles or geojson files, necessitates a process known as georeferencing [27]. Georeferencing involves a degree of cartographic knowledge, including the know-how of using mapping tools like ArcGIS or QGIS, selecting appropriate geospatial projections and reference systems, and aligning the image with precise geographic coordinates. This procedure can often be time-consuming and quite challenging. In HAIviz, we have streamlined these processes by allowing users to effortlessly drag-and-drop the image onto the interface and interactively label locations. The resulting map is then generated as an XML file, providing easy accessibility, data readability and updates.

HAIviz is a versatile tool suitable for various applications. It can be used for both quick, one-off visualisation projects or as an embedded platform to support ongoing surveillance programmes. For a simple and rapid visualisation, users can access HAIviz online, upload their input files directly, and create and download the visualisation results. Alternatively, users have the option to self-host HAIviz, configuring the platform with preloaded datasets including current and future projects. In this mode, users can add and update projects, thereby enabling real-time outbreak visualisation and reporting. This adaptable configuration is well-suited for clinical settings, empowering infection control professionals to provide regular updates and enhance infection control interventions.

HAIviz, operating as a standalone client-side single-page application, has a few noteworthy limitations, which should be taken into consideration: (1.) Limited Database Integration: HAIviz currently lacks the capability to integrate with databases, relying solely on user inputs or preloaded data for visualisation. Consequently, users cannot save or share their visualisation workspace within the system. It is recommended that users download the visualisation results prior to closing or refreshing their browser. (2.) Browser and Device Performance: The performance of HAIviz is contingent upon the user’s web browser and device. The user experience may vary based on the capabilities and performance of these tools. (3.) Lack of Analytical Tools: HAIviz currently serves as a visualisation endpoint, devoid of any analytical tools or pipelines. Consequently, users are required to undertake genomic epidemiological analysis using distinct tools prior to employing HAIviz for visualisation purposes. However, the absence of an integrated analysis toolkit makes HAIviz exceptionally adaptable, as it can seamlessly interface with pre-existing WGS analysis pipelines.

## Conclusion

HAIviz allows users to create an interactive genomic epidemiological visualisation dashboard using a web browser. The tool is easy to use and applicable to extend pathogen genomic surveillance both in general and healthcare settings. The tool is freely accessible online at https://haiviz.fordelab.com. The source code and the build application are available at https://github.com/nalarbudi/haiviz/ for self-hosting or offline use.

## Supplementary Data

Supplementary material 1
