# Supplementary material for: HAIviz: an interactive dashboard for visualising and integrating healthcare-associated genomic epidemiological data
Source: Microb Genom. 2024 Feb 15;10(2):001200. doi: 10.1099/mgen.0.001200 (PMC10926687; doi:10.1099/mgen.0.001200)
Supplement: Supplementary material 1 [file mgen-10-1200-s001.pdf]

Supplementary File S1: Supplementary methods, figure and tables

Supplementary methods

Demonstration dataset 1: The outbreak of carbapenem-resistant *Acinetobacter baumannii* in a Brisbane hospital.

The metadata input was created based on the previously published supplementary material 2 in Roberts et al. (1). The map input was generated by converting hospital ward layout image given by the author (Dr. Leah Roberts by personal communication) into HAIviz map and was also included as supplementary video in the study (1). For the transmission network input, a graph file in DOT format was created to convert the patients' transmission diagram from Roberts et al. (1).

Demonstration dataset 2: Genomic epidemiology of *Enterococcus faecium* ST78 in a Brisbane hospital

The metadata input was created based on our previously published study (2) listed in supplementary table S2. The patient stay input was generated based on the timeline of patient stay given in the study (2). The transmission network input was created using Outbreaker2 (3) based on genomic single nucleotide polymorphisms (SNPs) and sample collection dates on a cluster of closely related isolates defined using 20 SNPs cut-off. The resulting transmission graph was exported to a DOT-formatted file using igraph R package (4). Detailed transmission analysis was described in Permana et al. (2).

Demonstration dataset 3: The outbreak of *Klebsiella pneumoniae* ST15 and ST1559 in a Nepal hospital

The metadata input was created based on the previously published supplementary table S1 in Chung et al. (5). The map input was created by adapting the hospital layout image (Figure 1A in Chung et al. (5)) into HAIviz map. Phylogenetic tree input was reconstructed by comparing the published genomes from the study (5).

Performance test

The memory and CPU usage of HAIviz during the visualisation of sample datasets at sizes of 50, 250, 500, 750, and 1000 were recorded on the two major web browsers: Google Chrome and Mozilla Firefox. During the interactive visualisation session, the peak of memory and CPU usage was manually collected from the browser's built-in task

manager (Chrome: Task Manager, Firefox: address to `about:processes`). Each session was conducted in a separate new browser tab in private mode for each dataset size. The results were tabulated in the supplementary table S3 and were visualised in supplementary figure S1. The recording file documenting the test is available in the following link: https://www.youtube.com/watch?v=MVQKsO98mfo

Supplementary figure

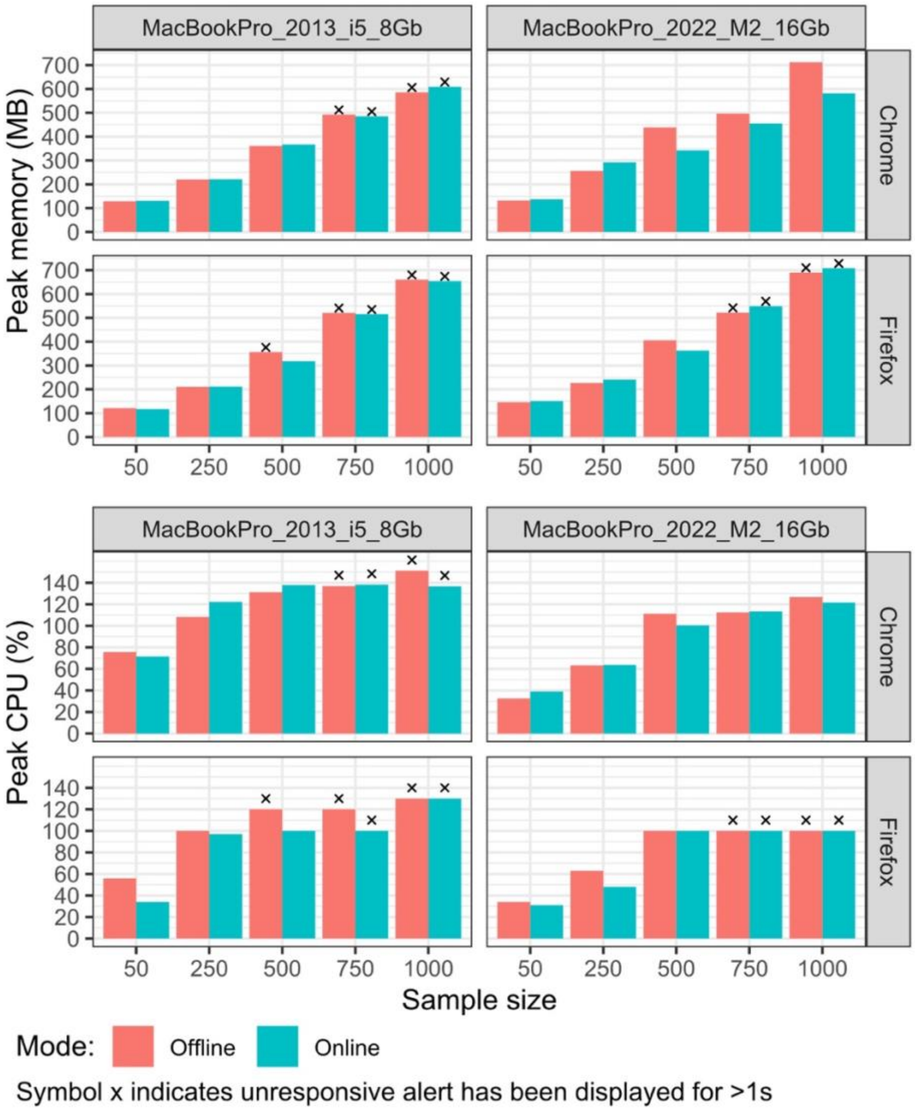

Figure S1: Peak memory and CPU usage during Haiviz visualization while loading five different dataset sizes (n=50, 250, 500, 750, and 1000) on Chrome and Firefox browsers. Tests were performed on an Apple MacBook Pro 2013 (RAM 8GB, Processor Intel i5) and a MacBook Pro 2022 (RAM 16GB, Processor Apple M2). Symbol x indicates unresponsive alert has been triggered and displayed for more than 1 second.

Supplementary tables

Table S1: Browser compatibility test

Page	Check point	Firefox v102.15	Chrome v116.0.5	Opera v102.0.4	MS Edge v120.0
Home	How it works video: loading and playing	☑	☑	☑	☑
Home	Quick start guide: linking to documentation	☑	☑	☑	☑
Home	Preloaded datasets: navigating to load data to dashboard	☑	☑	☑	☑
Home	Footer links: directing properly	☑	☑	☑	☑
Input	Links: directing to dashboard and documentation page	☑	☑	☑	☑
Input	Input placeholders: working input button	☑	☑	☑	☑
Input	Input placeholders: working drag-and-drop function	☑	☑	☑	☑
Input	Preloaded datasets: selecting and loading the data	☑	☑	☑	☑
Map Editor	Input placeholders: loading image and XML input	☑	☑	☑	☑
Map Editor	Add, update, and remove location button: working properly	☑	☑	☑	☑

Map Editor	Download XML map button: working properly	☑	☑	☑	☑
Dashboard	All windows: working draw functionality	☑	☑	☑	☑
Dashboard	All move, close, and setting buttons: working properly	☑	☑	☑	☑
Dashboard	All download buttons: working properly	☑	☑	☑	☑
Documentation	Quick start guide file: viewing and navigating properly	☑	☑	☑	☑
Documentation	All links: directing properly	☑	☑	☑	☑
Documentation	Download buttons: working properly	☑	☑	☑	☑
Documentation	Video: link is visible, and player is playing	☑	☑	☑	☑

Table S2: Summary of feature comparison between HAIviz and the existing genomic epidemiological visualisation web applications

Feature	HAIviz v1	Microreact v24	Pathogenwatch v21.2	Nextstrain
Focus and utility	Data visualisation for genomic epidemiology in healthcare settings.	Data visualisation and sharing for genomic epidemiology.	Global platform for pathogen genomic surveillance: genome upload and comparison, genotyping (cgMLST, ARG typing), visualisation, and data sharing.	An open-source pathogen surveillance, data visualisation and sharing platform, including bioinformatics and visualisation toolkit.
Online use/access	https://haiviz.fordelab.com	https://microreact.org/	https://pathogen.watch/	https://nextstrain.org
Availability for self-deployment or hosting	Compiled version of HAIviz is available at https://github.com/nalarbp/haiviz and can be downloaded and served by a web server for offline use or self-hosting	Not available	Not available	Components of Nextstrain such as augur and auspice, are available for the community in https://github.com/nextstrain
Main visualisation windows	Metadata table, non-geographic map, epidemic curve or sample collection timeline, phylogenetic tree, network, and Gantt chart	Metadata table, geographic map, phylogenetic tree, network, timeline and other customisable charts.	Phylogenetic tree, geographic map, cluster network, metadata table and sample timeline.	Phylogenetic time tree, geographic map, nucleotide diversity chart, summary of metadata.

Arrangement of visualisation windows	Dashboard-like: windows are resizable and freely arrangeable	Dashboard-like: windows are resizable and freely arrangeable	Limited grid-like: windows are resizable but not freely arrangeable	Limited grid-like: windows are arrangeable (in full or in fixed grid) but not resizable.
Map	Requires an XML file containing SVG image and location label.	Requires geographic coordinates (latitude and longitude) or ISO 3166 codes.	Requires geographic coordinates (latitude and longitude).	
Basemap creation and customisation	Users convert SVG image and add location labels interactively using a built-in Map Editor page to create the XML map.	Use geoJSON file for overlaying the boundaries using MapBox. The geoJSON creation requires third-party tool to georeferenced or to convert from the shapefiles.	Not supported	Not supported
Gantt chart	Displays a timeline of patient stays history. Patient id in y-axis and duration of stay in x-axis, coloured by the location.	Not supported. Microreact can create timeline and other charts but couldn't visualise patient stays or highlight their overlapping stay period.	Not supported	Not supported
Animation	Interactive and interlinked with other visualisation windows.			

<table>
<tr><td>Phylogenetic tree</td><td colspan="2"></td></tr>
<tr><td>Epidemic curve</td><td rowspan="2">Interactive and interlinked with other visualisation windows.</td><td>Not supported</td></tr>
<tr><td>Network</td><td>Transmission graph/network is overlaid to the map and interlinked with other visualisation windows</td></tr>
<tr><td colspan="3">Features of Microreact, Pathogenwatch, and Nextstrain were based on the version accessed in 18-September-2023</td></tr>
</table>

Table S3: Performance test

Test device	Browser	Resource	Mode	Sample size				
				50	250	500	750	1000
MacBookPro_2022_M2_16Gb	Firefox	Memory	Online	151	241	362	549 *	708 *
MacBookPro_2022_M2_16Gb	Firefox	Memory	Offline	146	227	406	522 *	690 *
MacBookPro_2022_M2_16Gb	Firefox	CPU	Online	31	48	100	100 *	100 *
MacBookPro_2022_M2_16Gb	Firefox	CPU	Offline	34	63	100	100 *	100 *
MacBookPro_2022_M2_16Gb	Chrome	Memory	Online	137	292	342	455	582
MacBookPro_2022_M2_16Gb	Chrome	Memory	Offline	132	256	439	496	712
MacBookPro_2022_M2_16Gb	Chrome	CPU	Online	39	63.7	100.3	113.3	121.6
MacBookPro_2022_M2_16Gb	Chrome	CPU	Offline	32.5	63.2	111.2	112.4	126.7
MacBookPro_2013_i5_8Gb	Firefox	Memory	Online	117	212	318	515 *	654 *
MacBookPro_2013_i5_8Gb	Firefox	Memory	Offline	121	211	356 *	521 *	660 *
MacBookPro_2013_i5_8Gb	Firefox	CPU	Online	34	97	100	100 *	130 *
MacBookPro_2013_i5_8Gb	Firefox	CPU	Offline	56	100	120 *	120 *	130 *
MacBookPro_2013_i5_8Gb	Chrome	Memory	Online	131	221	367	485 *	609 *
MacBookPro_2013_i5_8Gb	Chrome	Memory	Offline	129	220	361	492 *	586 *
MacBookPro_2013_i5_8Gb	Chrome	CPU	Online	71.5	122.2	138	138.3 *	136.7 *
MacBookPro_2013_i5_8Gb	Chrome	CPU	Offline	75.7	108.2	131.2	136.9 *	151.1 *

* Indicates unresponsive alert has been triggered and displayed for more than 1 second.

References

1. Roberts LW, Forde BM, Hurst T, Ling W, Nimmo GR, Bergh H, et al. Genomic surveillance, characterization and intervention of a polymicrobial multidrug-resistant outbreak in critical care. Microb Genom. 2021;7(3).
2. Permana B, Harris PNA, Runnegar N, Lindsay M, Henderson BC, Playford EG, et al. Using Genomics To Investigate an Outbreak of Vancomycin-Resistant Enterococcus faecium ST78 at a Large Tertiary Hospital in Queensland. Microbiol Spectr. 2023;11(3):e0420422.
3. Campbell F, Didelot X, Fitzjohn R, Ferguson N, Cori A, Jombart T. outbreaker2: a modular platform for outbreak reconstruction. BMC Bioinformatics. 2018;19(Suppl 11):363.
4. Nepusz GCaT. The igraph software package for complex network research. InterJournal. 2006;Complex Systems:1695.
5. Chung The H, Karkey A, Pham Thanh D, Boinett CJ, Cain AK, Ellington M, et al. A high-resolution genomic analysis of multidrug-resistant hospital outbreaks of Klebsiella pneumoniae. EMBO Mol Med. 2015;7(3):227-39.

Supplementary File S2: HAIviz's user manual

QUICK START GUIDE

HAIviz: Healthcare Associated Visualization Tool

v.1.0.0

Budi Permana

v.2024.01.15

Using HAIviz

Use it online

HAIviz is deployed at https://haiviz.fordelab.com for online use. Users can visit the web page using modern browsers (e.g., Google Chrome, Firefox, Microsoft Edge), drag and drop the input files, and instantly create visualisation dashboard.

All visualisation processes are performed locally in the user's browser with no data uploaded to the server, ensuring the safety of private data

https://**haiviz.fordelab.com/**

Tested and compatible with

Chrome provides the best experience.

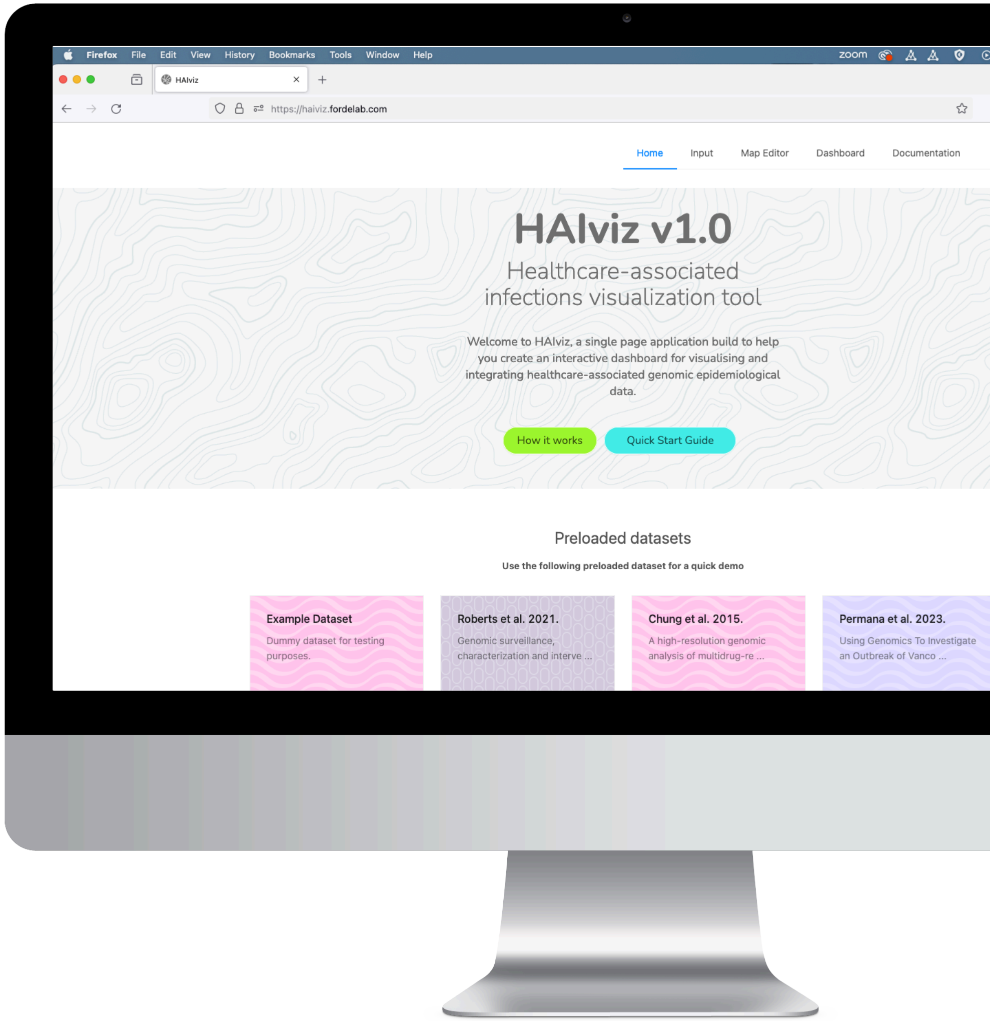

Self host or use it offline

Users also can use HAIviz offline by serving it through a static file server, such as, but not limited to `serve` Node.js package.

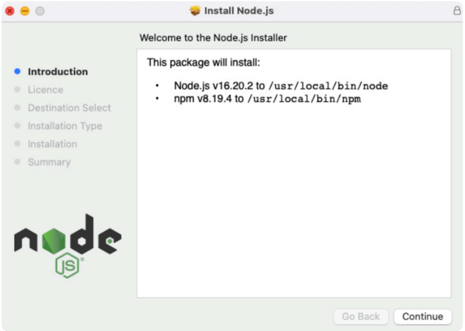

1. Install Node.js

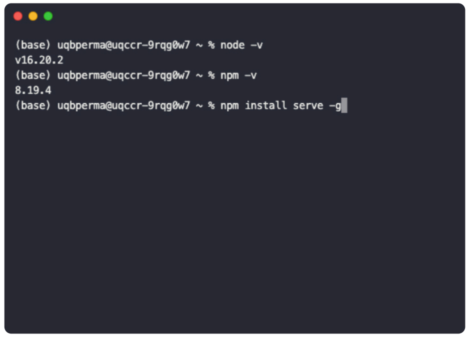

2. Install `serve` Node.js package

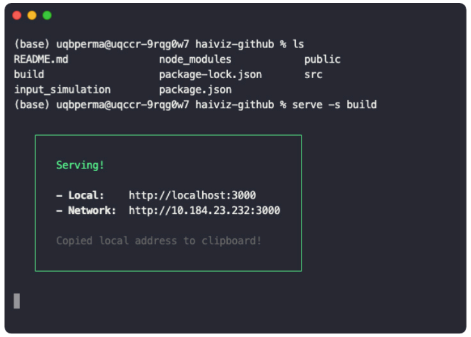

3. Serve the build directory

HAIviz is a single page application (SPA) visualisation tool that runs on the browser. Users can visualise and explore data by loading their input files or setting up preloaded datasets (can do it on the offline mode only).

Input Files

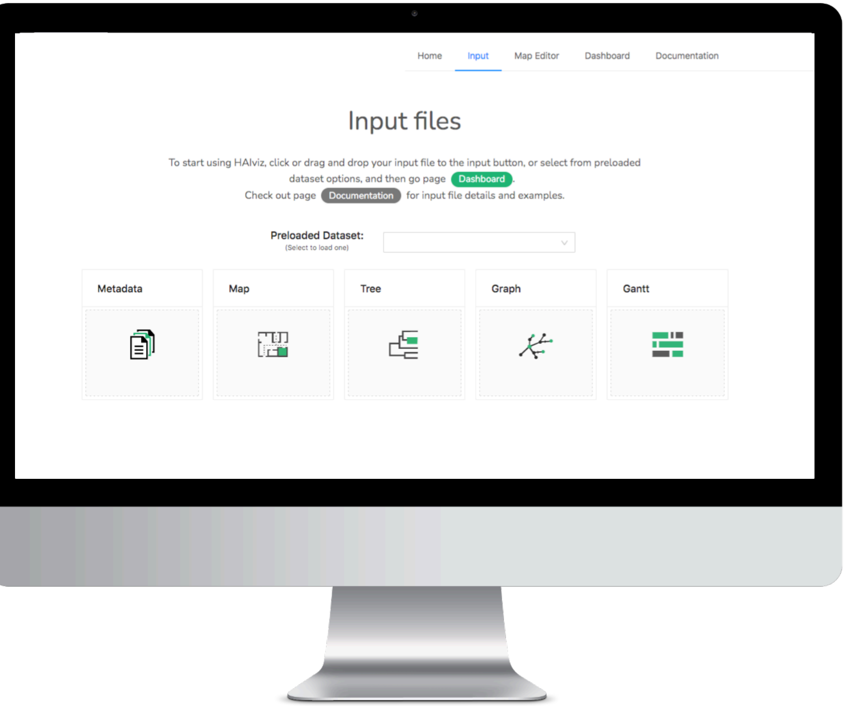

HAIviz is showing page *Input*. Users can click the file input loader or drag and drop the files into the input area. Input file will be parsed and validated, if file is valid, the window's icon in side-menu in page Dashboard will be **activated**.

Disabled

Activated

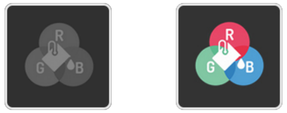

Window's icon

Metadata

A table contains information about the isolates or samples, written in CSV format. No duplicated records in column **id** and all dates must written in **ISO 8601 format (YYYY-MM-DD)**.

Mandatory columns (fixed header)			Other columns (optional, user-defined header)		Color columns (optional, [column]:color)	
**id**	**date**	**location**	**species**	**source**	location:color	species:color
Isolate1	2019-12-20	Location1	Species1	Patient	blue	#9e0142
Isolate2	2019-12-21	Location2	Species2	Environment	lightgreen	#c12949

Local map

An XML file contains SVG element and location indexes. This file is specific to HAIviz and can be created in page *Map Editor*.

```
<?xml version="1.0" encoding="UTF-8"?>
<haivizmap>
    <mapsvg>
        <svg
        xmlns="http://www.w3.org/2000/svg"
        id="haiviz-localmap-svg"
        width="1000" height="1000"
        viewBox="0 0 1000 1000" >

        <!-- The JPEG/PNG is embedded as
        the SVG element here-->

        </svg>
    </mapsvg>
    <mapdata>
        <location name="Location1" x="100" y="150"/>
        <location name="Location2" x="100" y="350"/>
    </mapdata>
</haivizmap>
```

Example of SVG attributes from a JPGE base image of 1000x1000px.

Location's x and y position from <mapdata> element.

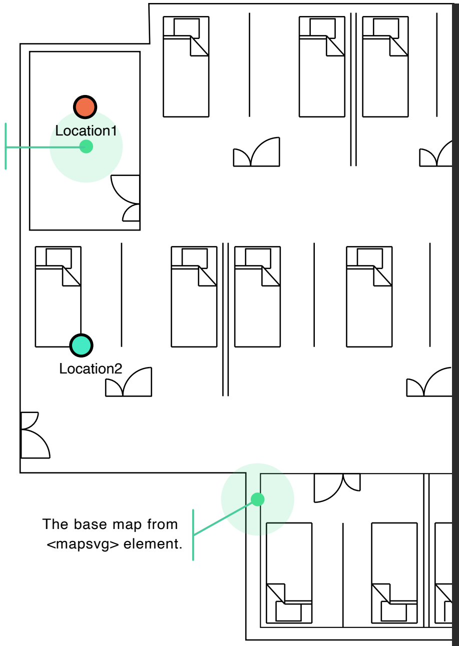

The base map from <mapsvg> element.

Input Files (cont.)

Phylogenetic tree

A Newick formatted phylogenetic tree file with taxa name (tip label) and branch length.

taxa name branch length

(Isolate4:0.5,(Isolate3:0.4375,(Isolate2:0.0625,Isolate1:0.1875):0.0625):0.0625);

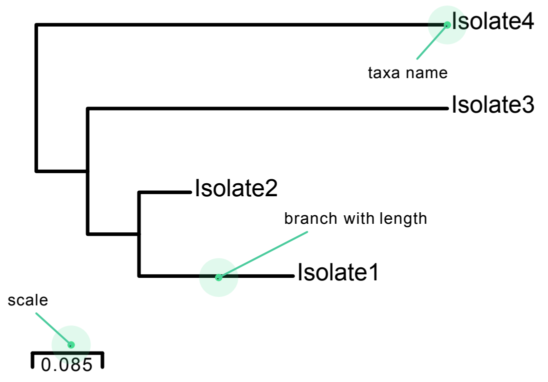

Network (graph)

A text file describing a graph object written in DOT language (**https://graphviz.org/doc/info/lang.html**). Using igraph R package you can export a network object into DOT format file with this following code: *write.graph(YourGraphObject, "myGraphInDOT.gv", format = "dot").*

directed graph object

node's attribute

node

```
digraph {
  0[name=Isolate1];
  1[name=Isolate2];
  2[name=Isolate3];
  0->1[color="red" weight=0.05 style="dashed"];
  1->0[color="black" weight=0.5 style="solid"];
  0->2[color="green" weight=1style="solid"];
  1->2[color="steelblue" weight=0.5 style="solid"];
  2->0[color="teal" weight=0.03 style="dashed"]
}
```

edge

edges's attributes

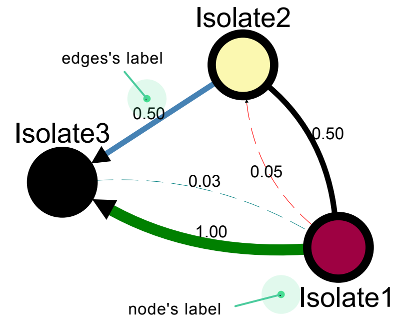

Movement timeline

A table file describing an individual movements (e.g. patient) from time to time at a single or multiple locations, written in CSV format. Column dates cannot empty and must written in **ISO 8601 format (YYYY-MM-DD),** and column *start_date* must be less than or equal to column *end_date*.

Mandatory headers and columns (Fixed header name) — **Optional**

pid	start_date	end_date	location	location_color
P1	2019-12-20	2019-12-25	Location1	#9E0142
P2	2019-12-21	2019-12-22	Location2	#49D1D8
P2	2019-12-23	2019-12-26	Location3	#FBF8B0

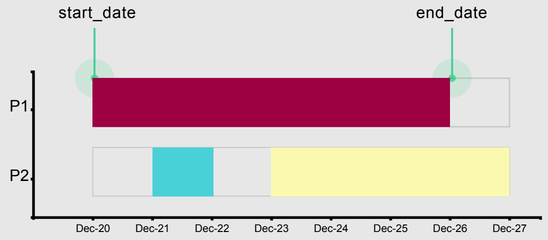

Dashboard Interface

Visualisation menu

Host all visualisation windows. Each icon will be activated as soon as visualisation from your input file is ready. Click on the icon to display the window.

Visualisation window

A window container where the interactive graphics are being rendered. The window can be individually moved, resized and closed.

Visualisation controller

Let you change the current state of visualisation display, such as, zoom in, zoom out and clear data selection.

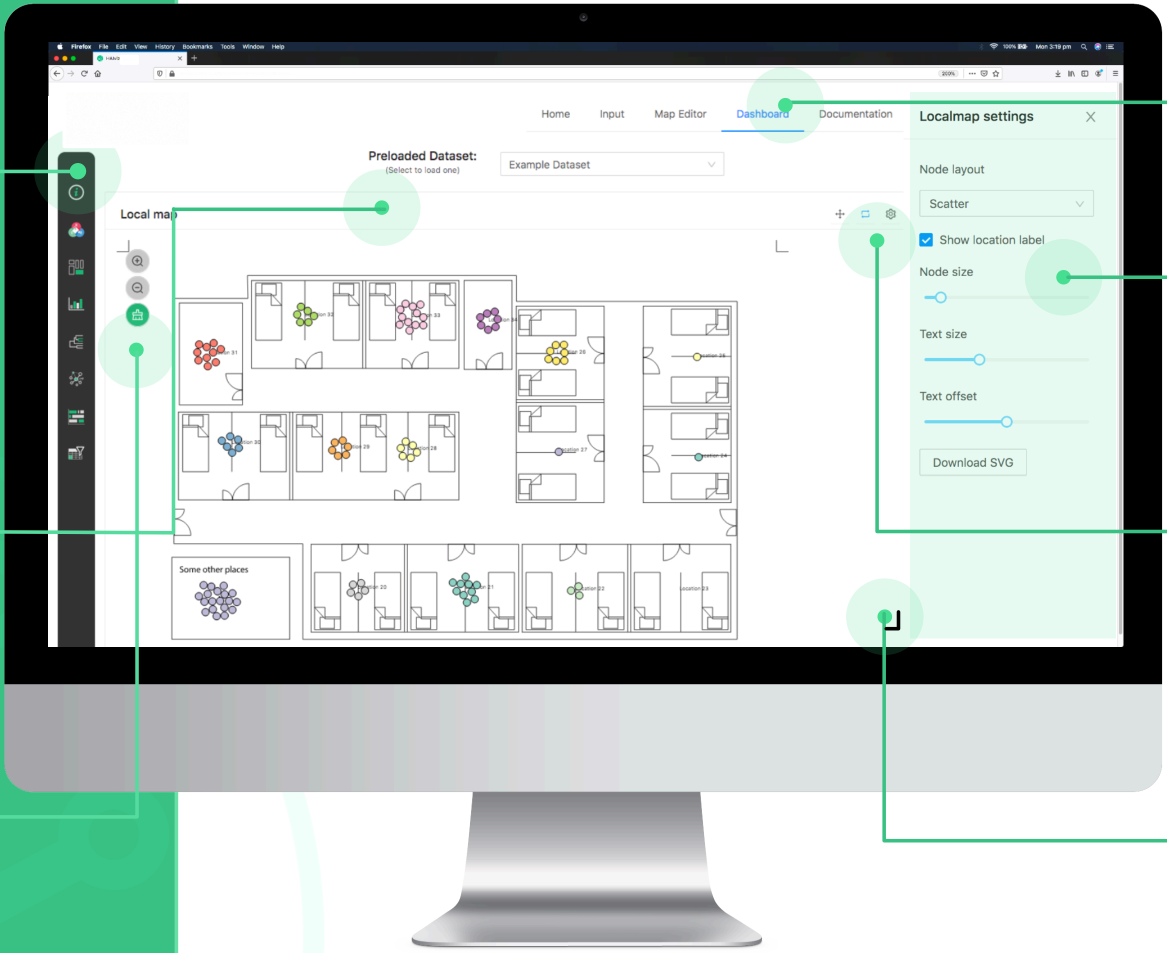

Navigation

The main application navigation to let you jump between pages.

Window settings

A side drawer provides you additional actions or settings related to the current window.

Window controller

(From left to right) Let you move the window, re-draw visualisation, open additional settings and close the window.

Resize controller

Let you change the current size of your visualisation window.

HAIviz is showing page Dashboard with map window displayed. Coloured nodes (circles) represent isolates clustered based in their location. Users can click, mouseover, zoom, pan, change the node layout, node size, location text size and download the the visualisation result.

Creating local map

HAIviz is showing page *Map Editor*. Users can start creating the map by loading an JPEG or PNG image. When the image file is loaded, users can add, rename, update or remove a location. A final map can be download for HAIviz use.

(!) - For a map to be downloadable, at least one map location must be added.
- Try open the downloaded Map (*haivizMap.xml*) in a text editor, you even can change the coordinates manually.
- The x and y coordiates follow the input base map image (e.g. x=0 and y=0 will be the very top-left of the image).

Location labeller

Let you enter and update the name of a location.

Zoom controller

Let you zoom in and zoom out the map.

Location logger

Let you add a new location and remove the selected location.

Location marker

To remove or update location name, users can click or drag to select the marker. Mouse over the circle will display the location label.

Map downloader

Let you download the map for later usage or load the map directly to HAIviz.

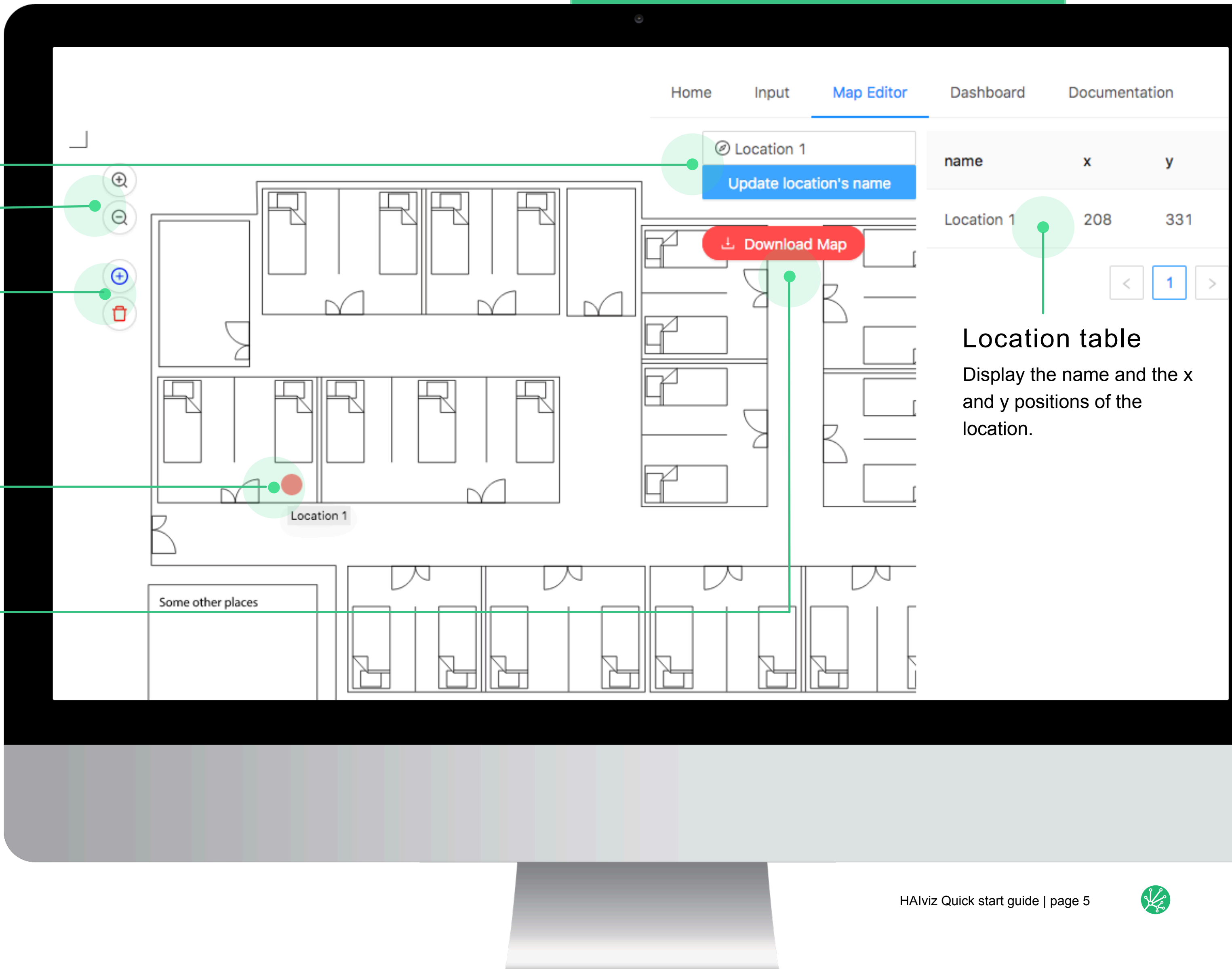

Location table

Display the name and the x and y positions of the location.

Five quick steps:

1. Add a location marker.
2. Move the marker to the desired coordinate.
3. Set the label for the marker.
4. Click the 'Update location's name' to update.

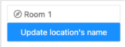

5. Repeat steps 1 to 4, then click Download Map.

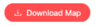

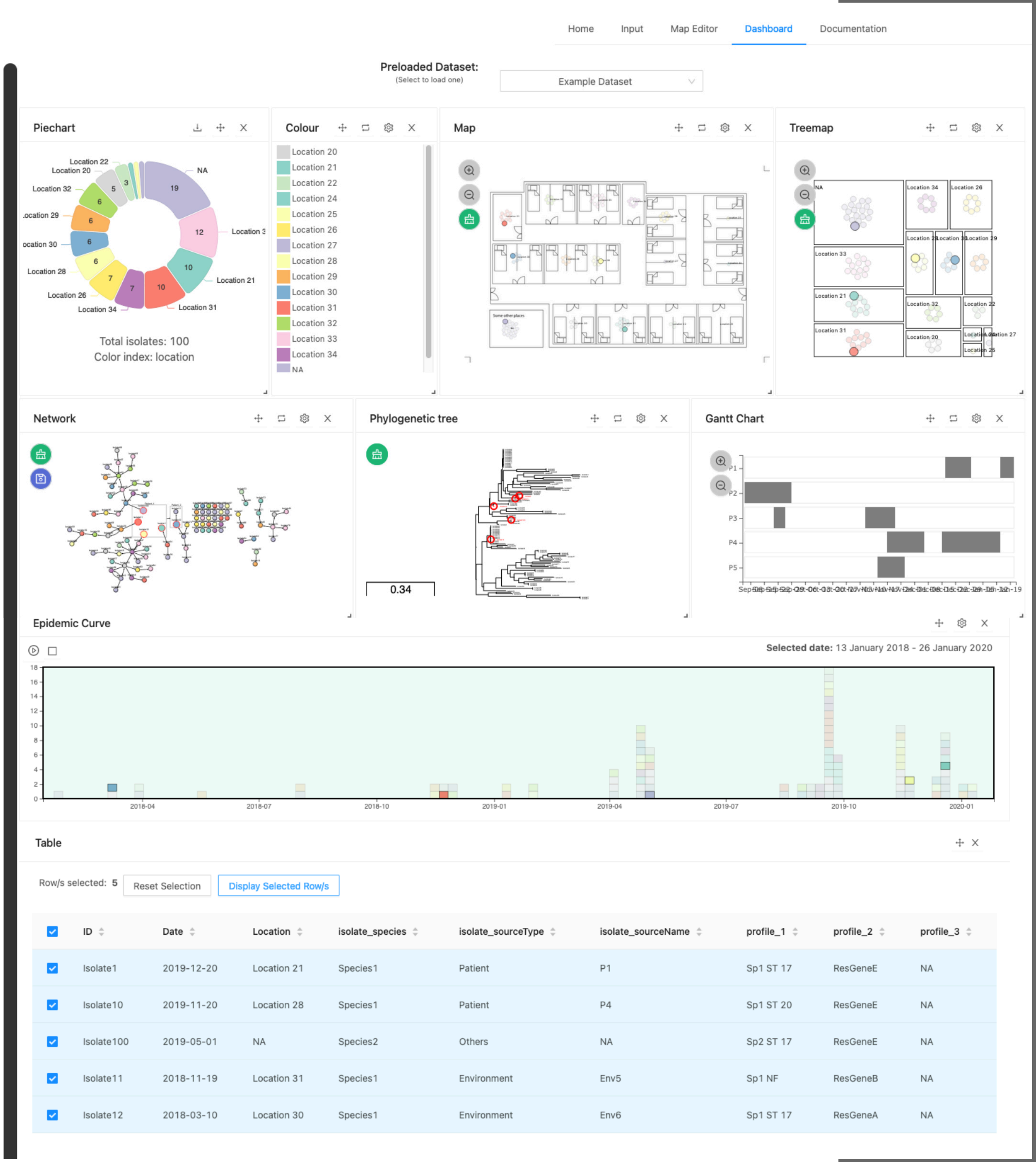

Interaction And Integration

- HAIviz is showing the integrated and interactive visualisation windows in page *Dashboard* created from an example dataset.

- To demonstrate integration functionality, five isolates were selected from the table window and were highlighted in the other active windows.

- Selection can also be performed in other windows, such as using an interactive brush on temporal distribution window to create animation.

- Image produced by HAIviz can be saved to an SVG format, enabling quick and flexible editing for report and publication.

Setting up preloaded dataset

When users self-host or use HAIviz offline, they can set up multiple preloaded datasets. This feature allows users to 'permanently' link their input files to HAIviz, avoiding the need to manually re-inputting their input files.

Example of directory tree of HAIviz build directory

```
build
 ├─ data
 │   ├─ preloaded
 │   │   └─ e0
 │   │       ├─ metadata.csv
 │   │       ├─ gantt.csv
 │   │       ├─ network.gv
 │   │       ├─ tree.nwk
 │   │       └─ map.xml
 │   └─ preloaded_dataset.json
 ├─ index.html
 └─ ...
```

1 Create a directory in the *preloaded* directory then add the input files.

2 Update the preloaded_dataset.json file

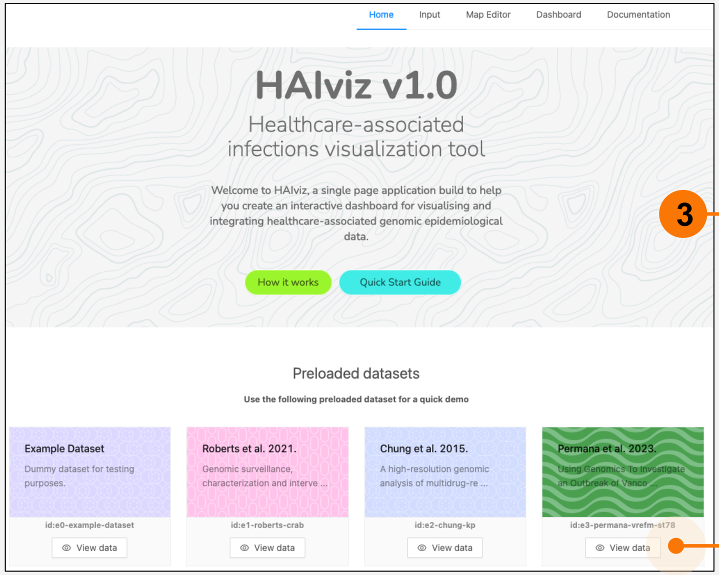

3 **Datasets is listed in HAIviz Home, Input, and Dashbord page.**

Click View data (in page Home) or select from the preloaded dataset dropdown button (in page Input or Dashboard) to load the all input files to HAIviz.

Example of preloaded_dataset.json file

```
{
  "data_list": [
   {
    "id": "e0-example-dataset",
    "name": "Example Dataset",
    "description": "Dummy dataset for testing purposes.",
    "metadata": "./data/preloaded/e0/metadata.csv",
    "map": "./data/preloaded/e0/map.xml",
    "tree": "./data/preloaded/e0/tree.nwk",
    "network": "./data/preloaded/e0/network.gv",
    "gantt": "./data/preloaded/e0/gantt.csv"
    }
   ],
 "description": ["This JSON file describes the preloaded datasets."]
}
```

An example R script to progamratically setup the preloaded datasets is given in the `input_simulation` directory in HAIviz repository : setup_preloaded_dataset.R.

THANK YOU

for reading this guide

Thanks to all awesome web frameworks and libraries run on the background, HAIviz is now up and running and available worldwide. The following are the core libraries used by HAIviz.

"@nivo/pie": "^0.61.1",
"antd": "^4.2.0",
"babel-polyfill": "6.26.0",
"cytoscape": "^3.17.0",
"cytoscape-cose-bilkent": "^4.1.0",
"cytoscape-fcose": "^2.2.0",
"cytoscape-spread": "^3.0.0",
"cytoscape-svg": "^0.3.1",
"d3": "^5.16.0",
"d3-array": "^2.4.0",
"d3-color": "^1.4.1",
"d3-delaunay": "^5.2.1",
"d3-fetch": "1.1.0",
"d3-scale-chromatic": "1.2.0",
"dotparser": "^0.4.0",
"export-to-csv": "^0.2.1",
"immutable": "3.8.2",
"install": "^0.13.0",
"jscrambler": "^5.5.18",
"jspdf": "^2.5.1",
"lodash": "^4.17.15",
"moment": "^2.25.3",
"moment-range": "^4.0.2",
"npm": "^6.14.9",
"phylocanvas": "^2.8.1",
"phylocanvas-plugin-export-svg": "^1.0.0",
"phylocanvas-plugin-scalebar": "^1.1.1",
"prop-types": "15.6.1",
"react": "^16.13.1",
"react-app-polyfill": "^2.0.0",
"react-color": "^2.17.3",
"react-dom": "^16.13.1",
"react-faux-dom": "4.1.0",
"react-file-drop": "^0.2.8",
"react-grid-layout": "0.16.6",
"react-measure": "1.4.7",
"react-pdf": "^4.2.0",
"react-player": "^2.7.2",
"react-redux": "5.0.7",
"react-router": "^5.1.2",
"react-router-dom": "^5.1.2",
"react-scripts": "2.0.0",
"recompose": "0.26.0",
"redux": "3.7.2",
"redux-immutable": "4.0.0",
"redux-promise": "0.5.3",
"reselect": "3.0.1",
"resize-observer-polyfill": "^1.5.1",
"svgsaver": "0.9.0",
"uuid": "^8.0.0",
"xml-formatter": "^2.0.1",
"xml-js": "^1.6.11"

React

Data-Driven Documents

Cytoscape

Ant Design

React-Grid Layout

Phylocanvas

HAIviz
Healthcare-Associated
Infections Visualization Tool
